# Microcontact Printing of Cholinergic Neurons in Organotypic Brain Slices

**DOI:** 10.3389/fneur.2021.775621

**Published:** 2021-11-17

**Authors:** Katharina Steiner, Christian Humpel

**Affiliations:** Laboratory of Psychiatry and Experimental Alzheimer's Research, Department of Psychiatry and Psychotherapy, Medical University of Innsbruck, Innsbruck, Austria

**Keywords:** cholinergic neurons, microcontact printing, nerve growth factor, organotypic brain slices, brain-on-a-chip

## Abstract

Alzheimer's disease is a severe neurodegenerative disorder of the brain, characterized by beta-amyloid plaques, tau pathology, and cell death of cholinergic neurons, resulting in loss of memory. The reasons for the damage of the cholinergic neurons are not clear, but the nerve growth factor (NGF) is the most potent trophic factor to support the survival of these neurons. In the present study we aim to microprint NGF onto semipermeable 0.4 μm pore membranes and couple them with organotypic brain slices of the basal nucleus of Meynert and to characterize neuronal survival and axonal growth. The brain slices were prepared from postnatal day 10 wildtype mice (C57BL6), cultured on membranes for 2–6 weeks, stained, and characterized for choline acetyltransferase (ChAT). The NGF was microcontact printed in 28 lines, each with 35 μm width, 35 μm space between them, and with a length of 8 mm. As NGF alone could not be printed on the membranes, NGF was embedded into collagen hydrogels and the brain slices were placed at the center of the microprints and the cholinergic neurons that survived. The ChAT+ processes were found to grow along with the NGF microcontact prints, but cells also migrated. Within the brain slices, some form of re-organization along the NGF microcontact prints occurred, especially the glial fibrillary acidic protein (GFAP)+ astrocytes. In conclusion, we provided a novel innovative microcontact printing technique on semipermeable membranes which can be coupled with brain slices. Collagen was used as a loading substance and allowed the microcontact printing of nearly any protein of interest.

## Introduction

Alzheimer's disease is a neurodegenerative disorder leading to a progressive decline in cognitive and intellectual functions without a clear causative event. The cardinal pathological features of Alzheimer's disease (AD) are extracellular β-amyloid (Aβ) depositions (plaques) and intraneuronal hyperphosphorylated tau inclusions (neurofibrillary tangles (NFTs)). This is accompanied by chronic inflammation as a contributor to the neurodegenerative processes observed in AD but also in PD (1, 2). There is only a weak correlation between cognitive decline, Aβ plaques, and NFTs, but the density of neocortical synapses strongly correlates with all three, indicating that synaptic loss is the major correlate of cognitive impairment in AD (3). The main populations of cholinergic neurons are located in the basal forebrain: the nucleus basalis of Meynert (nBM) and medial septum. They provide projections to the entire neocortex and hippocampus, synthesizing and releasing acetylcholine (4, 5). It has been demonstrated that the selective degeneration of this transmitter-specific neuronal population in the nBM is a major hallmark in AD (6, 7). Indeed, the decline of cholinergic neurons directly correlates with a decrease in cognitive and intellectual functions (8). However, so far, it is still unclear if the cell death of cholinergic neurons is a primary event in AD or is caused by the dramatic deposition of Aβ plaques or tau NFTs in the cortex and hippocampus.

The survival of basal forebrain cholinergic neurons is dependent on the classical nerve growth factor (NGF) (9–11). The NGF is synthesized in the target area of cholinergic neurons: the cortex and hippocampus. As NGF is a target-derived neurotrophic factor, it is endocytosed by the cholinergic nerve fibers and retrogradely transported to the somata in the nBM/septum where an NGF-dependent transcriptional program is activated (12). Two different cell-surface receptors, as well as the state of NGF (unprocessed pro-form or mature form), determine the activity and function of NGF. Mature NGF has a higher affinity for the tropomyosin receptor kinase A (trkA) receptor promoting survival and growth, whereas pro-NGF preferentially binds to neurotrophin receptor p75^NTR^-mediating apoptotic signaling (13). Increasing evidence supports that the NGF function is affected in AD (14) as an imbalance between the NGF/TrkA-mediated survival signaling and pro-NGF/p75^NTR^-mediated apoptotic signaling may occur. Moreover, the TrkA-dependent retrograde transport of NGF could be impaired for the Aβ and tau pathology and cholinergic neurons are not able to take full advantage of the NGF (12). Thus, NGF may also have potential therapeutic implications in AD, as therapeutic strategies aim to deliver NGF directly into the brain (15–17).

Organotypic brain slice cultures bridge the gap between *in vitro* cell cultures and *in vivo* animal experiments. In contrast to homogenous single cell cultures, in organotypic brain slice cultures, the complex three-dimensional architecture of the brain is preserved, simulating more *in vivo*-like situations (18–20). Additionally, organotypic brain slice cultures permit markedly reducing the number of animal experiments. As the cell death of cholinergic neurons is the central hallmark of AD, organotypic brain slices are a potent tool to study the neurodegeneration of cholinergic neurons in AD. The presence of cholinergic neurons in organotypic slices is verified in the septum/hippocampus (4) and the nBM, the latter extensively studied in our lab (9, 21–24). It is well-established that NGF is required to maintain cholinergic neurons in organotypic brain slices (9, 11, 21, 25). In this study, NGF supports the survival of cholinergic neurons in nBM organotypic brain slices when applied to a medium (9, 21). We also developed a model, where NGF was locally applied directly onto brain slices using collagen hydrogels (1, 11, 26).

To take it a step further, we aimed to immobilize the growth factors in a pattern next to the slice enabling guided neural fiber outgrowth. Therefore, we took advantage of a high-resolution (sub-μm range) patterning technology called microcontact printing (μCP). The μCP technique is referred to as soft lithography and is widely used to immobilize proteins onto a variety of different background materials (27, 28). However, μCP seems to be a straightforward technique for printing clear and even plasma-activated surfaces, but this does not apply to pored membranes. Currently, only a single study describes printing onto membranes using heavy-ion etched polycarbonate membranes (29). Printing onto pored membrane inserts, however, has not been demonstrated so far. Recently, we were successful in printing antibodies onto pored membranes and coupled these antibodies with the growth factor glial-cell line-derived neurotrophic factor (GDNF) to stimulate the nerve fiber growth of dopaminergic neurons (1).

The present study aimed to establish the microcontact printing of the protein NGF on semipermeable 0.4 μm pore membranes and to couple them with organotypic brain slices of the nBM. To print the NGF, collagen was used as a loading biomaterial. We will demonstrate that cholinergic neurons will survive and grow along the NGF microcontact prints.

## Materials and Methods

### Organotypic Brain Slices of the nBM

Organotypic chopper brain slices were prepared as reported in detail in our lab (21). Briefly, postnatal day 8–10 C57BL/6 wildtype mouse pups were rapidly decapitated and their brains were dissected under sterile conditions. The nBM was dissected according to our scheme (21) and 300 μm slices were chopped on a McIlwain Tissue chopper (Mickle Laboratory Engineeering Co. LTD, Loughborough, England. The brain slices were carefully transferred to an Isopore^TM^ 0.4 μm pore PC membrane (HTTP02500, Merck Millipore, Burlington, Massachusetts, United States) with μCP lanes (see below). These membranes were transferred onto semipermeable 0.4 μm pore cell culture inserts (PICM03050, Merck Millipore) which were placed into 6-well-plates (Greiner Bio-One, Kremsmünster, Austria). Each well-contained 1.1 ml of sterile-filtered culture medium (50% MEM/HEPES (Gibco, Carlsbad, California, United States), 25% heat-inactivated horse serum (Gibco/Lifetech, Austria), 25% Hanks' solution (Gibco), 2 mM NaHCO_3_ (Merck, Austria), 6.5 mg/ml glucose (Merck, Germany), and 2 mM glutamine (Merck, Germany), at pH 7.2)). The brain slices were cultured at 37°C with 5% carbon dioxide (CO_2_) for 2–6 weeks and the culture medium was changed once a week. The slices attached to the membranes were flattened and became transparent. The brain slices were cultured with or without 100 ng/ml of recombinant anti-mouse β-NGF (50385-MNAC, Sino Biological, Germany) in a culture medium. After 2–6 weeks, the slices were fixed for 3 h at 4°C in 4% paraformaldehyde and stored at 4°C in 10 mM of phosphate-buffered saline (PBS) until use. All the experiments conformed to the Austrian guidelines on the ethical use of animals and were in line with the reduce, refine and replace (3Rs) rule as all efforts were made to reduce the number of animals. In fact, from one mouse pup, we were able to generate 50–100 brain slices dependent on the area and purpose. All animal experiments were defined as “Organentnahme” according to the Austrian laws. For this project, 47 mouse pups were used.

### Preparation of Collagen Hydrogel Solution

Collagen hydrogels were prepared as we have described in detail (11). As a crosslinker, 4S-Star-polyethyleneglycol succinimidyl succinate (4S-StarPEG) (JKA7006-1G, Sigma, St. Louis, Missouri, United States) was used. Two mg/ml sterile bovine collagen solution type I (804592-20ML, Sigma) was linked with 0.4 mM of 4S-StarPEG in PBS at pH 7.4. The Collagen- polyethylene glycol hydrogel (PEG) solution was loaded with a recombinant anti-mouse β-NGF (50385-MNAC; Sino Biological) in a final concentration of 10 ng/μl of NGF or fluorescent Alexa-546 anti-goat antibodies (final concentration: 20 ng/μl). An equal volume of PBS was added to generate the control collagen hydrogel microcontact prints. During the handling, all the components were kept on ice to prevent pre-mature gel formation. Approximately 100 μl of collagen hydrogel ink solution ($\hat{=}$ 1 μg NGF) was immediately applied onto the μCP stamp (see below).

### Microcontact Printing

**Figure 1:**
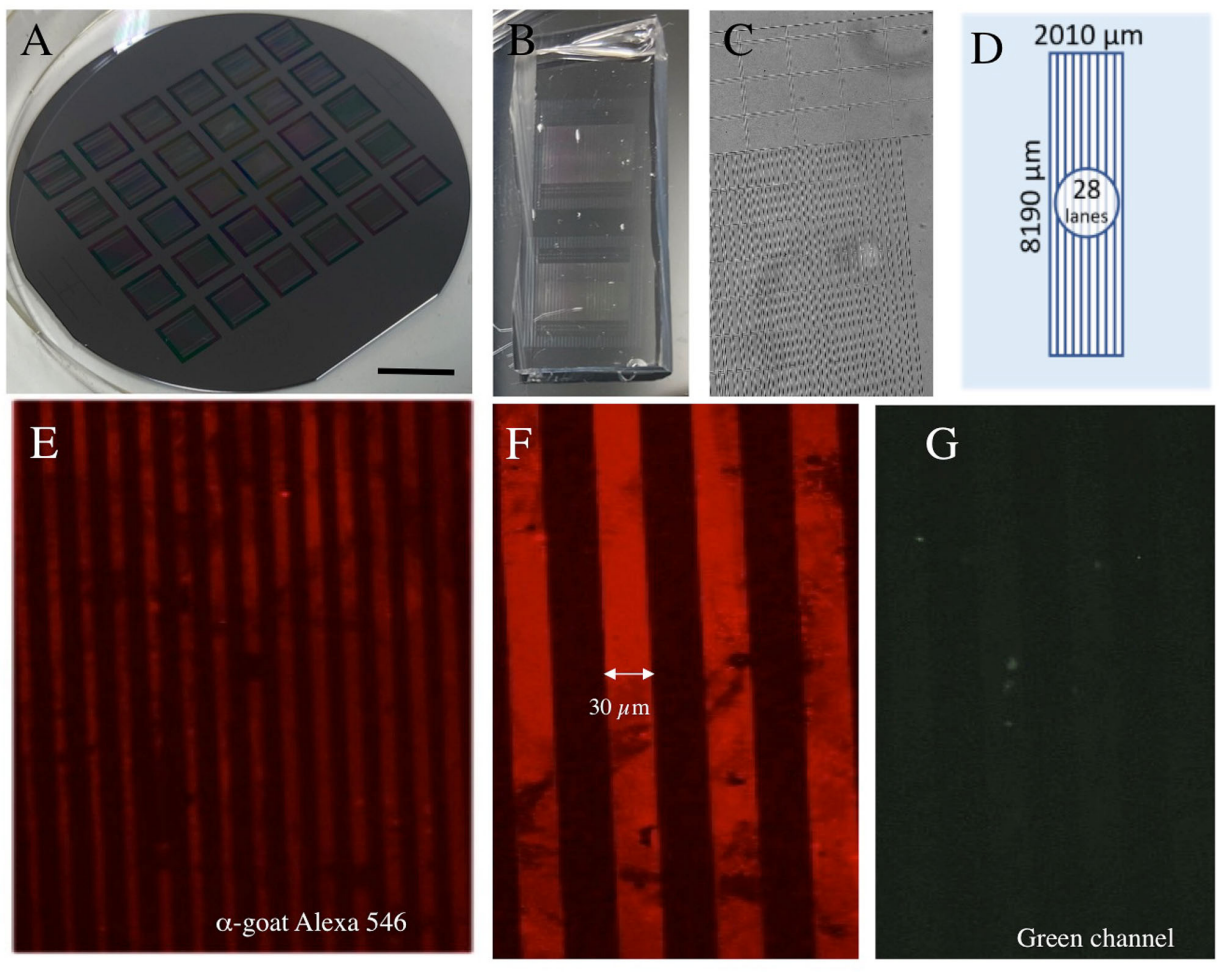
Characterization of microcontact printing (μCP). **(A)** The “silicon wafer master mold” with 6 × 5 stamp masters. **(B)** Two polydimethylsiloxanes (PDMS) stamps. In **(C)** the PDMS stamp is seen under a phase-contrast microscope. A typical stamp has a length of 8 mm and a width of 2 mm including 28 lanes **(D)**. The microcontact printing was set up with the printing of an anti-goat Alexa-546 antibody showing red lines **(E)** with 30 μm size and space **(F)**, which is not seen in the green channel **(G)**. Scale bar in A = 1.75 cm **(A)**, 0.5 cm **(B)**, 250 μm **(C)**, 140 μm **(E)**, 52 μm **(F,G)**.

The *silicon wafer master mold* (Figure 1A) was a kind gift from Jenny Emnäus and Janko Kajtaz (Department of Biotechnology and Biomedicine, DTU Bioengineering, Technical University of Denmark), and has been used previously in a common publication (1). The master mold has been fabricated by photolithography using a silicon oxide layer (4.7 μm) and is described elsewhere.

The *Micropatterned stamp fabrication* was performed as we have previously described (1). Polydimethylsiloxane is highly suitable for protein adsorption and transfer due to its hydrophobic surface. The polydimethylsiloxane (PDMS) prepolymer (SylgardTM 184 Silicone elastomer kit, 001004176976, Dow) arrived in two components. The elastomer curing agent was carefully mixed with an elastomer base solution in a concentration of 1:10. The surface relief of the PDMS stamps was formed by casting and curing liquid PDMS against the micropatterned silicon wafer master mold (Figures 1B,C). The raised and lowered regions of the silicon wafer were mirrored into the stamps and the final patterns were defined. After being left to cure overnight at 60°C, the solid PDMS was peeled off the mold and the stamps were cut to size with a scalpel for further use.

The μ*CP* was performed similarly as reported from our lab (1) but modified. Microcontact printing uses an elastomer stamp which adsorbs the “ink” solution and transfers it to a surface at a very high resolution. Approximately 100 μl of the liquid collagen hydrogel ink solution (loaded with PBS, NGF, or anti-goat Alexa-546 antibodies) was applied directly onto the micropatterned stamp. To distribute the ink solution equally, a coverslip was placed on top. After 15 min of incubation at 37°C, the coverslip was removed and used to carefully strike off the remaining ink solution, once with and once against the lanes of the pattern. The excess solution on the borders of the pattern was removed using filter paper without touching the printing surface and was left to air dry for a minute. As soon as the stamp was completely dry, the ink solution was transferred to the semipermeable membrane by pressing it on with an 18 grams weight for 60 min at room temperature. The position of the stamp was marked with four small dots of permanent marker for the arrangement of the slices. Then, the weight and the stamp were carefully removed from one corner. The membranes were sterilized under a UV light for 20 min, equilibrated with the slice medium, and placed on the inserts before arranging the brain slices. To optimize the NGF μCP, a dose- (1,000–100–10 ng NGF) and time- (0–14 days) dependent experiment was performed. For all the μCP experiments, we used the Isopore^TM^ 0.4 μm pore PC membrane (HTTP02500, Merck Millipore). We also compared μCP on Omnipore^TM^ 0.45 μm PTFE membranes (JHWP02500, Merck Millipore) and LCR 0.45 μm PTFE membranes (FHL02500, Merck Millipore).

### Immunohistochemistry

Immunohistochemistry was performed as previously described under free-floating conditions (21, 30). This method allows the antibody to penetrate from both sides during incubation, enhancing the sensitivity of the staining. The outgrowth processes were considered to be the single-cell layers on the membrane. First, the fixed brain slices were incubated in 0.1% Triton-PBS (T-PBS) for 30 min at room temperature with soft shaking. After incubation, the brain slices were washed 3 × 3 min with PBS and subsequently blocked in 20% horse serum/0.2% bovine serum albumin (BSA)/T-PBS for 30 min while shaking. Following the blocking, the brain slices were incubated in 0.2% BSA/T-PBS with primary antibodies, namely, choline acetyltransferase (ChAT) (Merck AB144P, 1:750), glial fibrillary acidic protein (GFAP) (Merck AB5541, 1:2000), laminin (Sigma L9393; 1:500), p75^NTR^ NGF receptor (Abcam ab52987, 1:750), microglial Iba-1 (Wako 019-19741; 1:500), microtubule-associated protein-2 (MAP-2), Chemicon MAB3418; 1:500), or NGF (Cedarlane MC51, 1:250) for 48 h at 4°C. After incubation, the brain slices were washed 3 × 3 min with PBS and incubated with the corresponding green fluorescent Alexa-488 (or red fluorescent Alexa-546) secondary antibodies (1:400 in 0.2% BSA/T-PBS) for 1 h at room temperature while shaking. The secondary antibodies were: anti-goat for ChAT, anti-chicken for GFAP, anti-rabbit for laminin, p75^NTR^, NGF, Iba-1, and anti-mouse for MAP-2. The brain slices were counterstained with the blue fluorescent nuclear dye, 4′,6-diamidino-2-phenylindole (DAPI) (1:10,000 diluted in T-PBS), for 30 min. The brain slices were washed again with PBS before being mounted on glass slides with Mowiol (Carl Roth, Karlsruhe, Germany). The staining was visualized with a fluorescence microscope (Olympus BX61, Olympus Corporation, Shinjuku City, Tokyo, Japan) and Openlab software 5.5.0 (Improvision, Germany). For the co-stainings, the slices were washed 3 × 5 min with PBS before the application of the second primary antibody. Some sections were stained with the chromogenic DAB.

### Data Analysis and Statistics

Quantitative analysis was performed blinded under the microscope. The ChAT+ nBM neurons were recognized when clear cytoplasmic staining with at least one neuronal fiber extension and a definable nucleus was observed. The slices were excluded if the number of ChAT+ neurons was <20/slice. As two chopper slices were placed side by side in the center of the μCP, the outgrowths projecting upwards and downwards were separately quantified. The number of ChAT+ processes was evaluated along the NGF μCP and they were counted as single nerve fibers, nerve fiber bundles, thick nerve fiber networks, and outgrowths with and without ChAT+ cells. To evaluate the length of the outgrowth, a picture was taken under the Olympus BX61 fluorescence microscope and the pixel number of the outgrowth was measured using the OpenLab 4.0.4 software connected to a MAC computer (Apple, Cupertino, California, United States). The pixel number was measured from the slice border to the very top of the outgrowth. The values obtained were then converted to micrometers based on a scale bar. To evaluate the intensity of the NGF μCP, the optical density (OD) was measured. Therefore, a picture was taken with 1 s exposure time and 10×-magnification and afterward was converted into greyscale. Then a rectangle of 30 × 100 pixels was chosen within a printed lane and transferred to Photoshop Elements 2.0 (Adobe, San Jose, California, United States). After inverting the picture, the OD mean was taken from the histogram and corrected for the background. The OD measurement was repeated 3 times per print. The sample size (*n*) pertains to the number of analyzed mice. All the values are given as mean ± SEM. Statistical analysis was performed by one-way ANOVA with a subsequent Fisher least significant difference (LSD) *post-hoc* test, where *p* < 0.05 represents significance.

## Results

### Characterization of μCP Using Antibodies

To characterize the method, we first performed μCP on a fluorescent antibody, as established in our lab. Using the wafer master mold (Figure 1A), PDMS stamps (Figure 1B) were produced with a size of 2 × 8 mm (Figures 1C,D) and 28 lanes (Figure 1D). The printing of an anti-goat Alexa-546 antibody showed several red lines under the fluorescence microscope (Figure 1E), with a line width of 30 μm and a space of 30 μm (Figure 1F). The staining was not seen in the green channel, showing the specificity of μCP (Figure 1G).

### Characterization of NGF μCP With Collagen Hydrogel Solution

**Figure 2:**
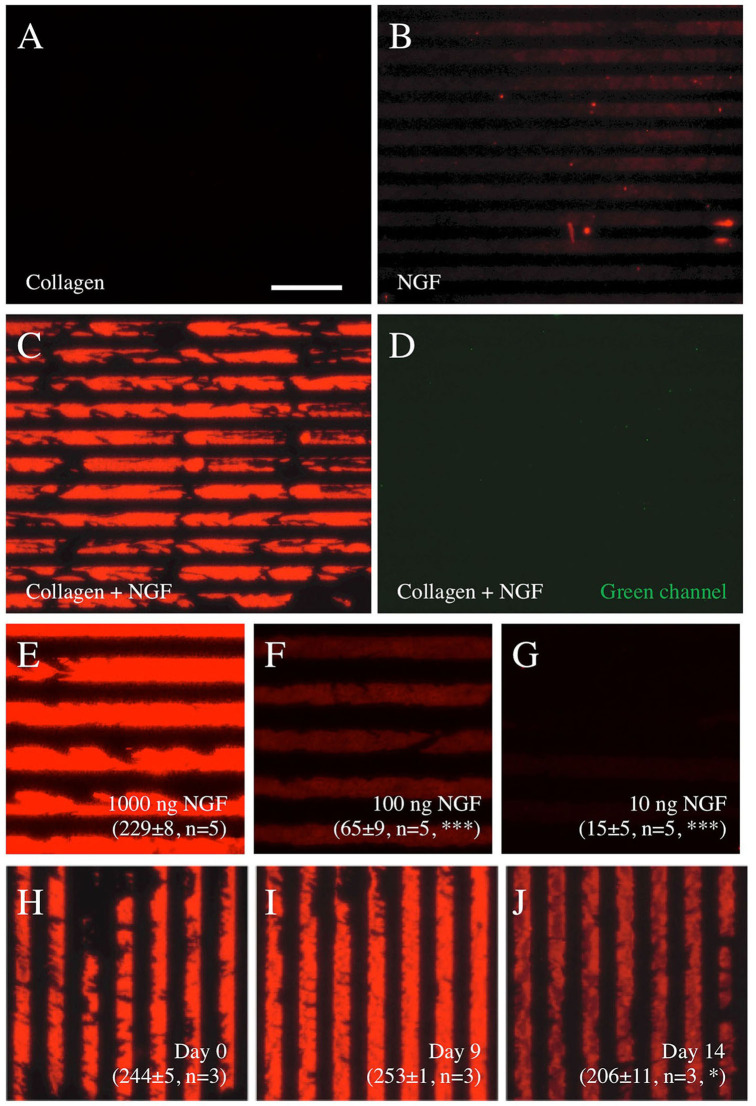
Characterization of nerve growth factor (NGF) μCP on semipermeable membranes. Printing of collagen alone **(A)** or NGF alone **(B)** does not show any positive staining, however, NGF loaded into collagen hydrogel solution shows a typical printing when stained with Alexa-546 anti-NGF antibodies **(C)**. The staining is specific, as it is not seen in the green channel **(D)**. A concentration experiment shows that printing with 1,000 ng NGF per stamp **(E)** gives the best results, while 100 ng **(F)** and 10 ng **(G)** NGF do not show good printing. The stability of the prints (1,000 ng NGF loaded into collagen hydrogel solution) in the medium is shown in **(H–J)**, with strong bands after printing [day 0, **(H)**], no change after 9 days in the medium **(I)**, and a significant decrease after 14 days in the medium **(J)**. The values in **(E–J)** give the optical density of the printed pattern from 0 (white) to 255 (black) corrected for the background and measured by computer-assisted imaging. The values are presented as mean ± SEM, with the number of independent experiments (*n*) and statistical differences (*p < 0.05; ***p < 0.001) with ANOVA and Fisher least significant difference (LSD) *post hoc* test. Scale bar in A = 175 μm **(A–D)**, 102 μm **(E–G)**, 175 μm **(H–J)**.

While it was easy to microprint antibodies, the μCP of NGF alone did not give a positive signal (Figure 2B). To establish the μCP of a protein (NGF) on the semipermeable (0.4 μm pore) membranes, we used a well-established technique to produce collagen hydrogels. While collagen alone gave the background only (Figure 2A), the μCP of the NGF loaded in the collagen hydrogel solution showed a strong and clear print and many lines after staining with an Alexa-546 anti-NGF antibody (Figure 2C). Again, no signal was seen in the green channel (Figure 2D). To optimize the NGF μCP, different concentrations of NGF were tested, and 1,000 ng NGF per load gave the best results (Figure 2E, 229 ± 8 optical density, *n* = 3). The μCP of 100 ng NGF (Figure 2F, 65 ± 9 optical density, *n* = 3) and 10 ng NGF (Figure 2G, 15 ± 5 optical density, *n* = 3) significantly showed a weaker signal. To demonstrate the stability of the NGF μCP, the membranes were incubated in a “slice medium” for up to 2 weeks and the stability did not change after 9 days (Figure 2I) but markedly decreased after 14 days (Figure 2J), compared with the control (Figure 2H). After 6 weeks of incubation, the NGF μCP was no longer detectable (8 ± 1 optical density, *n* = 3). Compared with the Isopore membranes (HTTP022500), the μCP of NGF onto the Omipore membranes (JHWP02500) and LCR PTFE membranes (FHLC02500) was 3.3× less effective (data not shown).

### Characterization of Slices in Contact With μCP

**Figure 3:**
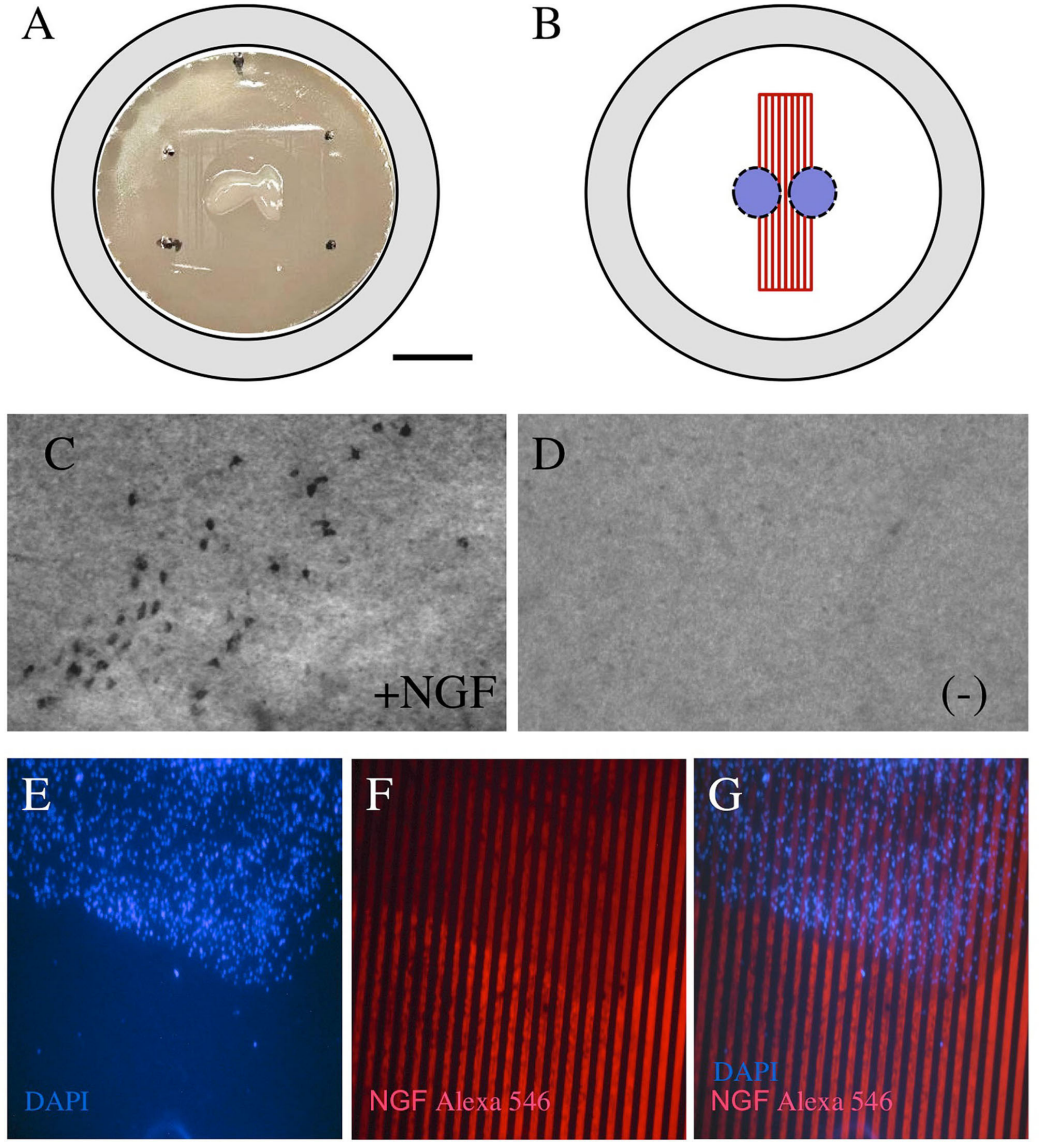
Coupling of brain slices to microcontact prints. Chopper brain slices (300 μm) were prepared from the basal nucleus of Meynert (nBM) and cultured on semipermeable membrane inserts **(A)**. The slices were coupled with the μCP of NGF **(B)**. After 2 weeks in culture, the cholinergic neurons were stained for choline acetyltransferase (ChAT) and many cholinergic neurons survived when incubated with 100 ng/ml NGF in the medium **(C)**, while no cholinergic neurons were visible when incubated without NGF **(D)**. The brain slices were stained for nuclear 4′,6-diamidino-2-phenylindole (DAPI) [**(E)**, blue] and were connected to the NGF μCP (stained by anti-NGF Alexa-546, red), showing that the slice grows directly in connection with the μCP. Scale bar in A = 500 μm **(A)**, 130 μm **(C,D)**, 250 μm **(E–G)**.

The brain slices from postnatal mice were incubated on semipermeable membrane inserts with pre-prepared μCP (Figure 3A). The NGF lines were printed on semipermeable extra membranes and the brain slices were connected to the μCP (Figure 3B). Cholinergic neurons were stained for ChAT and several ChAT+ nBM neurons survived when incubated with 100 ng/ml NGF in the medium (Figure 3C) but not without NGF (Figure 3D). The brain slices were stained with the blue fluorescent nuclear dye DAPI (Figure 3E) and connected to the NGF μCP stained with an Alexa-546 antibody (Figure 3F). Figure 3G shows that the blue fluorescent slice was directly connected to the red fluorescent NGF μCP lines.

### Characterization of Cholinergic Nerve Fibers Along the NGF μCP

**Figure 4:**
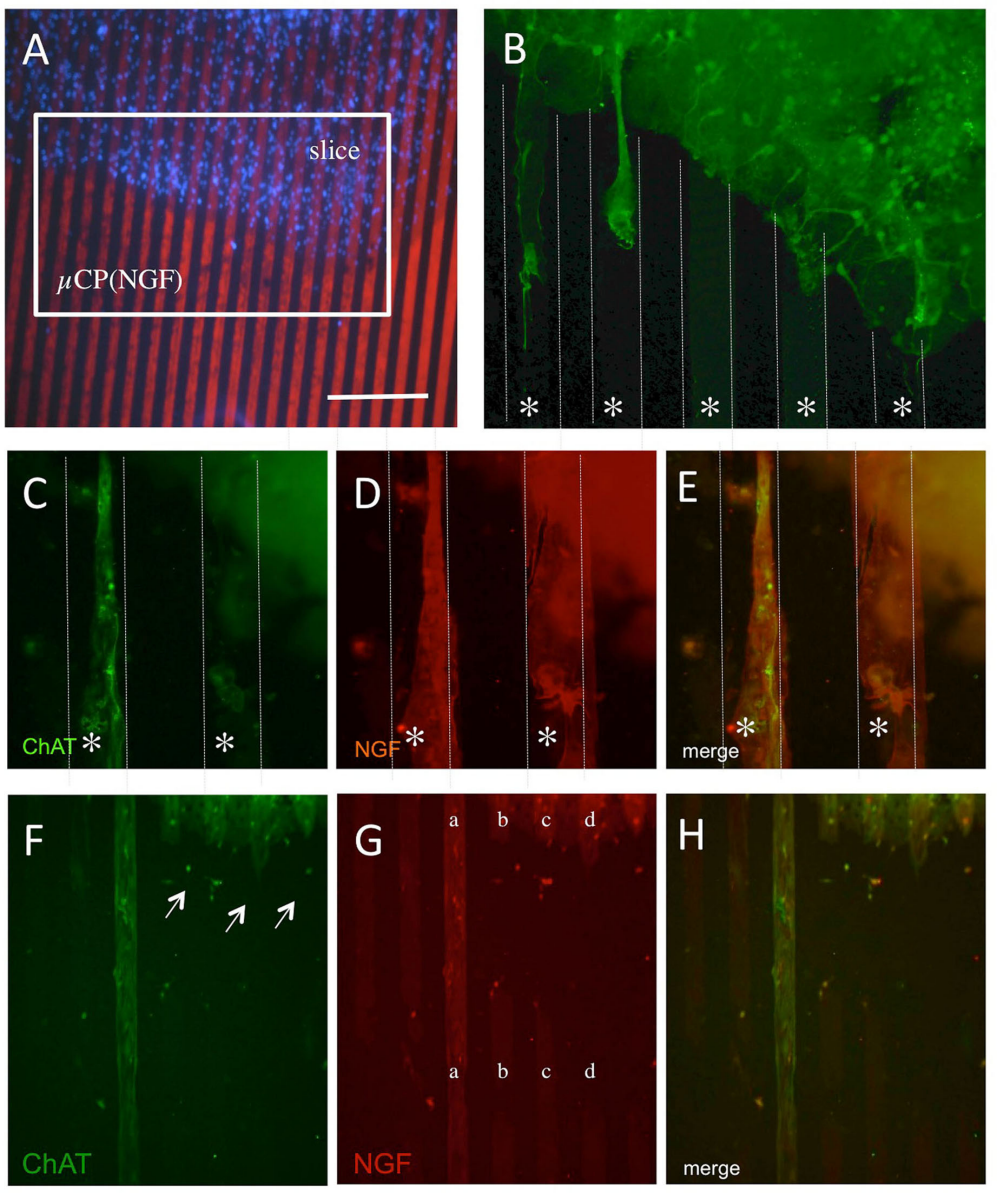
The outgrowth of cholinergic neurons from the brain slices coupled with NGF microcontact prints (μCP). Brain slices (300 μm thick) of the nBM were prepared and connected to the NGF μCP, incubated for 1 week with 100 ng/ml of NGF in the medium, and then further for 2 weeks without NGF. The slices were fixed and stained for ChAT and Alexa-488 (green) and the ChAT processes were evaluated at the borders of the slices **(A)**. **(B)** The outgrowth of ChAT+ neurons along the μCP (white lines, star *). Three typical outgrowths are seen, thin axons, but also thicker processes. **(C–E)** A typical example of an outgrowth of ChAT+ nerve fibers [**(C)**, green, Alexa-488], co-stained with an anti-NGF antibody [**(D)**, red, Alexa-546]. **(E)** Shows the merged picture and that the ChAT+ nerve fibers grow along the NGF μCP. **(F–H)** The selectivity of growth along the μCP. **(G)** An example of an incomplete printing (lanes b–d), while on a complete print, (lane a) the ChAT+ processes are seen [**(F)**, arrows]. Quantitative analysis shows the outgrowth of 24 ± 2 ChAT+ nerve fibers per slice with a length of 286 ± 35 μm after 3 weeks in culture. Ten brain slices were prepared from five animals. Scale bar in A = 270 μm **(A)**, 60 μm **(B)**, 54 μm **(C–E)**, 108 μm **(F–H)**.

In the next step, we characterized the ChAT+ processes growing out of the brain slices (Figure 4A). Three weeks after incubation on NGF μCP membranes, several ChAT+ cholinergic neurons were visible and several ChAT+ processes grew toward the NGF μCP (Figure 4B). The ChAT+ extensions were clearly nerve fibers (Figure 4C) that grew along the NGF μCP (Figures 4D,E). As an additional proof of specificity, Figures 4F–H show a μCP that was not homogenously printed and not intact (Figure 4G, lines b-d). The processes stopped growing on the not intact or damaged areas (Figure 4F, arrows), while the processes grew a long distance on the intact long printed lines (Figure 4F, line a; and Figure 4H).

The number of processes outside the slice and along the NGF μCP was 24 ± 2 (*n* = 12) processes per slice and the average length of the processes was 286 ± 35 μm (*n* = 12) evaluated after 3 weeks in culture. The outgrowth slightly decreased after 6 weeks in culture: 16 ± 2 (*n* = 12; *p* < 0.01) processes/slice and 205 ± 19 μm (*n* = 12, not significant) length of fibers.

**Figure 5:**
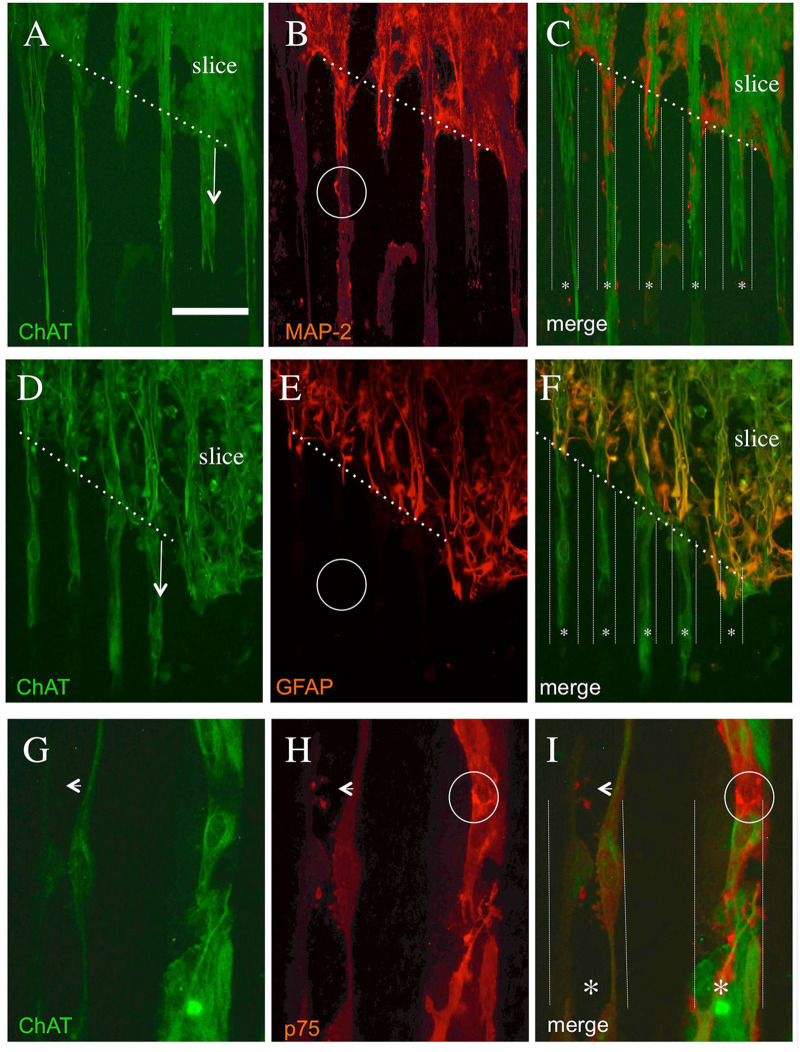
Co-localization of cholinergic neurons stained with ChAT **(A,D,G)** and with microtubule-associated protein-2 [MAP-2, **(B)**] or glial fibrillary acidic protein [GFAP, **(E)**] or the low-affinity NGF receptor p75^NTR^ **(H)**. Brain slices were cultured on NGF μCP for 3 weeks, fixed, and stained for ChAT (Alexa-488, green) and MAP-2, GFAP, or p75^NTR^ (Alexa-546, red). The merged pictures **(C,F,I)** show the orientation of the slices, the borders (white thick dotted lines), and the suggested μCP lanes (white small dotted lines with a star *). **(A)** The ChAT+ processes (six lanes) grew out of the slice (arrow) along the μCP. **(B)** The MAP-2+ processes which fully co-localized with the ChAT+ processes and display a differential expression intensity [see as an example the white circle in **(B)**]. **(D)** ChAT+ processes (4 lanes) growing out of the slices (arrow) along the μCP. **(E)** shows intense GFAP+ staining within the brain slices, more extensive at the borders representing reactive astrogliosis, but nearly no immunopositive processes outside the slices [see the white circle in **(E)**]. **(G)** The immunostaining of two selected ChAT+ processes along a μCP, which fully co-localizes with the p75^NTR^ neurotrophin receptor **(H)**. The small arrow in **(G–I)** point to strong differential p75^NTR^ immunoreactive varicosities (probably synaptic processes) within the ChAT+ processes, but also strong neuronal immunoreactive areas [white circle in **(H,I)**] are found. Note that all these stainings do not come from the same slice, but from four to six brain slices prepared from two to five animals. Scale bar in A = 180 μm **(A–F)**, 36 μm **(G–I)**.

The co-localization experiments showed that the neuronal MAP-2+ immunoreactivity (Figure 5B) partly overlapped with the cholinergic ChAT+ staining (Figures 5A,C), though MAP-2 was less strongly expressed than ChAT. The overlaps were seen within the slice, but also in the ChAT+ processes (Figure 5C). An intense GFAP staining was observed in the brain slices (Figure 5E) which did not show any staining in the ChAT+ processes (Figures 5D,F). The co-staining of cholinergic ChAT+ processes (Figure 5G) with the low-affinity NGF receptor p75^NTR^ (Figure 5H) shows strong immunoreactivity partly in the cholinergic processes (Figure 5I).

### Re-organization Within the Brain Slices

**Figure 6:**
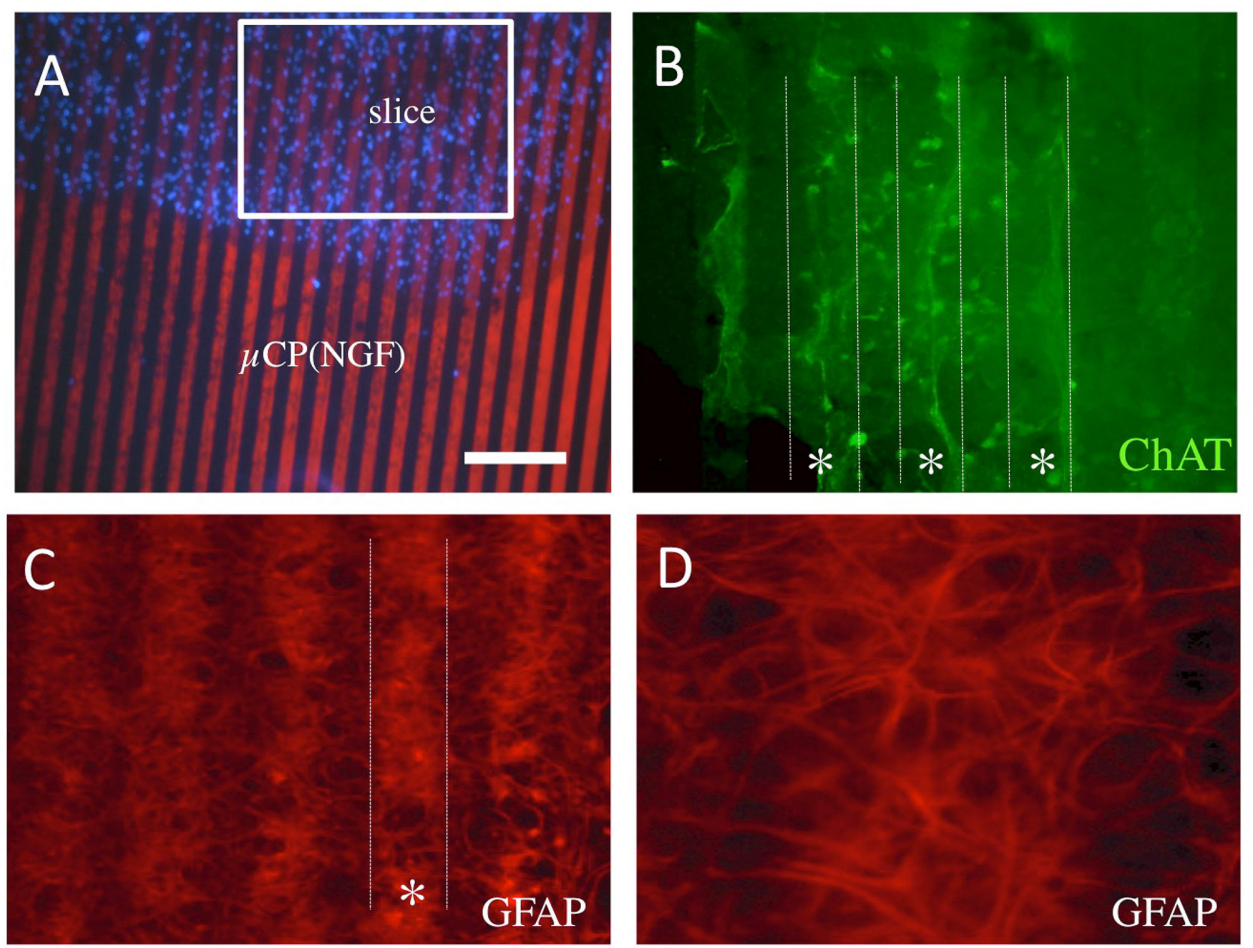
Re-organization within the slices placed onto the NGF μCP **(A)**. Note some form of the re-organization of ChAT+ cholinergic neurons and fibers **(B)** along the μCP [**(B)**, dotted lines, star *]. The GFAP+ cells and processes re-organized in the slice and grew on the NGF μCP [**(C)**, dotted white line] leaving some empty areas in-between **(D)**. For each representative two to four brain slices were prepared from two to five animals. Scale bar in A = 210 μm **(A)**, 50 μm **(B,C)**, 25 μm **(D)**.

For the methodological issues, the brain slices were placed directly into the NGF μCP (Figure 6A). Besides the axonal growth, we also could see a strong re-organization of cells within the brain slices. There appeared a slight re-organization of the ChAT+ neurons and fibers along the μCP within the slices (Figure 6B). The GFAP+ cells and extensions were organized along the NGF μCP in the slices, leaving some blank lines in between (Figures 6C,D). No such re-organization was seen for the microglial Iba1+ cells (data not shown) or laminin+ vessels (data not shown).

### Migration of Cells Along the μCP

**Figure 7:**
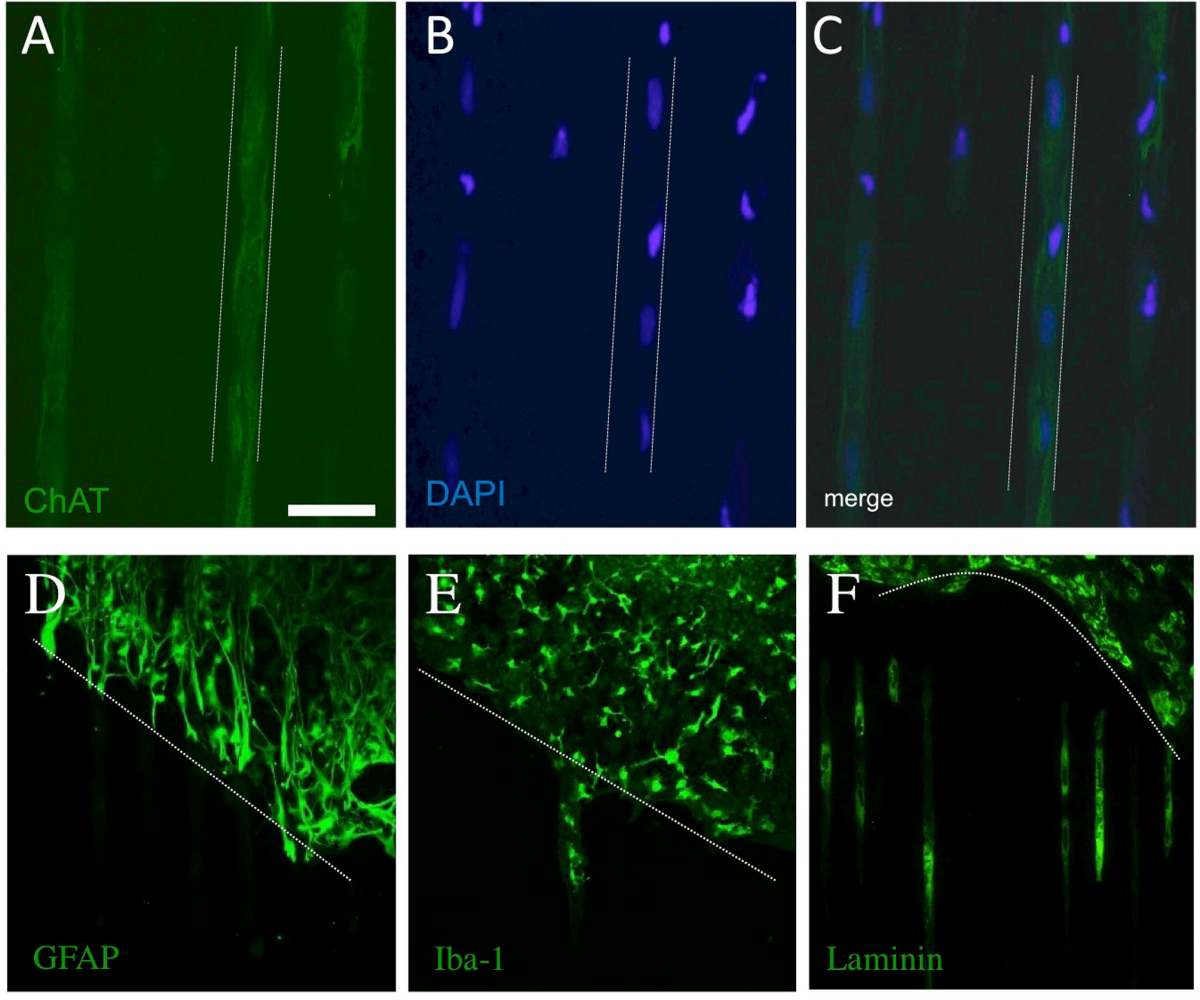
Migration of cholinergic neurons (choline acetyltransferase, ChAT+), astrocytes (glial fibrillary acidic protein, GFAP+), microglia (Iba-1+), and endothelial cells (laminin+) along the collagen μCP. The dotted lines in **(A–C)** indicate the μCP lanes and in **(D–F)** the borders of the slices are shown by a dotted line. The ChAT+ processes grew along the μCP(NGF) lanes **(A)**, and blue fluorescent DAPI+ nuclei are seen in these processes **(B,C)**, suggesting the migration of cholinergic neurons. Glial fibrillary acidic protein was highly expressed within the slices, but no processes were seen outside **(D)**. Iba-1+ **(E)** as well as laminin+ **(F)** cells migrated along the collagen μCP. Twenty brain slices were prepared from five animals. Scale bar in A = 100 μm **(A–C)**, 200 μm **(D–F)**.

Besides the numerous processes outside of the slices, we also observed that cells migrated along the μCP lines. Figures 7A–C shows an example wherein DAPI+ nuclei are found along a μCP (Figure 7B), co-localizing with the ChAT+ stainings (Figures 7A,C). No GFAP+ astrocytes migrated outside the brain slices (Figure 7D), but microglial Iba1+ (Figure 7E), as well as more laminin+ endothelial cells (Figure 7F), migrated along the collagen μCP.

## Discussion

In the present study, we show for the first time that we can microprint the protein NGF onto semipermeable membranes when loaded into the biomaterial collagen. We could connect a brain slice to the NGF μCP and provide data that cholinergic processes grew along the NGF μCP and that astrocytes re-organized in the brain slices.

### Organotypic Brain Slices as a Tool to Study Axonal Growth

Organotypic brain slice cultures represent a physiologically relevant three-dimensional *ex vivo* model [see our review (20)]. As the main architecture of the cells is preserved, it allows the *in vitro* investigation of cellular and molecular processes in the brain areas of interest. The group of Gähwiler was the first who succeeded in culturing organotypic brain slices utilizing the roller-tube technique (31). This technique was modified, maintaining organotypic brain slices cultured on semipermeable membranes (19). In our lab, we used the “chopper” technique and have extensively studied the cholinergic neurons of nBM (9, 11, 21–24, 30), dopaminergic neurons (1, 30, 32), or serotonergic neurons (33). A major benefit of organotypic brain slice cultures is the opportunity to perform co-culture experiments, which allows the study of two or more related brain areas. Dopaminergic nerve fiber growth between two co-slices has been reported by several research groups (34–36) including ours (1, 32), enabling the investigation of dopaminergic neurons concerning Parkinson's disease. Similarly, we showed that the nerve fibers of cholinergic neurons from the nBM grow toward the cortex in organotypic brain slice co-cultures (37). Organotypic brain slice cultures were prepared from postnatal day 8–10 brains as the tissue and cell survival is high in this timeframe (37). The older the donor animals (>14 days postnatal), the more tissue and cell death occur in culture. The brains of younger donor animals exhibit a looser texture and morphology, as they are more immature. The slices were stained at the earliest after 2 weeks *in vitro*, as they need this period to completely flatten from 300 to ~100 μm (20). The grade of flattening is not only an important parameter of macroscopic cell survival but also increases the quality of immunostaining and microscopic analysis. The thicker the slices, the less antibodies are able to diffuse deep into the slices and the images may appear more blurry. However, it is an advantage that we performed all immunostainings free-floating, as antibodies are then able to penetrate the slice from both sides improving image quality.

### Microcontact Printing of Pure NGF Alone

Microcontact printing allows cellular engineering by printing proteins of interest in defined stripes and it is a simple and efficient method to pattern different surfaces with a wide range of proteins. In the present study, we aimed to microprint NGF alone onto semipermeable membranes to study cholinergic nerve fiber growth *in vitro*. First, we reproduced a μCP of fluorescently labeled antibodies onto a semipermeable membrane to demonstrate that printing still works in our study (1). The antibodies preferentially attach to the hydrophobic PDMS stamp and are excellently transferable, thereby confirming that μCP is an efficient method for protein transfer to semipermeable membranes. The μCP of NGF alone onto semipermeable membranes, however, was not efficient. We have already observed this problem before, trying to microprint GDNF protein alone onto semipermeable membranes (1). To study the outgrowth of dopaminergic nerve fibers, we printed anti-GDNF antibodies onto the semipermeable membrane and loaded them with the GDNF protein (1). As far as we know, the microprint of pure NGF onto semipermeable membranes or any other substrate has not been published yet and the microprint of pure NGF alone onto semipermeable membranes was not effective in our study. This may have several reasons, namely, the (a) inefficient adsorption of NGF to the surface of the PDMS stamp, (b) inefficient transfer of NGF to the semipermeable membrane, (c) diffusion and loss of NGF through the pores of the semipermeable membrane, (d) disruption of the NGF molecule structure during printing procedure, or (e) blockade of the anti-NGF antibody recognition region preventing its detection.

### Collagen as a Carrier to Microprint NGF

Collagen is the main component of connective tissue, accounting for almost one-fourth of the total body protein in humans (38), and in the nervous system, it supports cell differentiation, attachment, migration, proliferation, and survival (39). Therefore, collagen is highly suitable as a raw material for tissue-engineered scaffolds providing a biocompatible, biodegradable, non-toxic, and versatile possibility to mimic both the structural and biological properties. Our laboratory has already acquired a lot of experience in working with collagen (40). So far, we were able to load collagen hydrogels in organotypic brain slice cultures with NGF (11), fibroblast growth factor-2 (26), and GDNF (1) taking advantage of the PEG crosslinking system. In contrast to our earlier study (11), we aimed not only to protect the cholinergic neurons in the nBM via NGF application but also to initiate axonal growth in defined directions. Therefore, we microprinted a collagen hydrogel solution loaded with NGF to immobilize NGF in the pattern next to the slice enabling guided neural fiber outgrowth. In the present study, we succeeded in microprinting NGF loaded in collagen onto 0.4 μm pore semipermeable membranes. To our knowledge, collagen has never been microprinted together with any other protein onto semipermeable membranes. There are two reports showing techniques in applying NGF in combination with collagen, however, it was “molecular printed” to a collagen gel (41) and “3D bioprinted” (42). In our present study, we verified specificity *via* immunostainings for NGF with fluorescent antibodies. Furthermore, the quantitative evaluation showed that the μCP of NGF was dependent on the amount of NGF loaded. Thus, the microprinting of NGF onto semipermeable membranes using collagen hydrogel solution was shown by this study for the first time. This method is easy, fast, cheap, and versatile as nearly any protein can be printed, which is shown by our preliminary experiments, e.g., we can microprint a 50 kDa tau protein but also a 4 kDa β-amyloid peptide (Supplementary Figure 1).

### Cholinergic Neurons and NGF-Induced Axonal Growth Along NGF μCP

Cholinergic neurons are located in the nBM and the medial septum providing projections to the entire neocortex and hippocampus, respectively (4, 5). The neurotransmitter acetylcholine has a relevant impact on memory and a loss of acetylcholine in AD directly correlates to memory loss (6, 7). Cholinergic neurons were immunohistochemically stained for the enzyme ChAT as this enzyme is exclusively expressed in the cytosol of cholinergic neurons and we have extensive experience with such stainings (21, 37). Cholinergic neurons express the neurotrophin receptor p75^NTR^ which almost entirely co-localizes with ChAT in the nBM, as well as the medial septum (43). The NGF is a target-derived neurotrophic factor and it is well-known that NGF is essential for the viability of cholinergic neurons (9–11). In the present study, we confirmed previous work and showed that the μCP of NGF supports the survival and subsequent nerve fiber growth of nBM neurons in organotypic brain slices.

Commonly, the extracellular matrix proteins poly-lysine, fibronectin, and laminin are microprinted onto glass slides or Petri dishes before neuronal cell populations are seeded (44–46). As those proteins are known to support neural cell growth and differentiation, this approach is frequently used to study neurons in cell culture. Taking advantage of the same approach, others succeeded in μCP semaphorins (47), ephrins (48), and netrins (49) onto glass slides or plastic dishes. All of them are molecules known to effectively direct axon guidance. In the present study, we used the target-derived neurotrophic factor NGF to guide cholinergic nerve fibers along the μCP lines.

To connect the organotypic brain slices to the μCP, freshly prepared slices were carefully placed onto the μCP membrane. The slices were positioned in the center of the μCP, which enabled us to investigate neuronal growth in two directions per slice. The most difficult point was to ensure that the slices had obtained the desired orientation on the μCP, as the μCP itself was hardly or not visible at all during the arranging procedure. The marks on the membrane assisted us in assuming the localization of the μCP and we were able to positively connect the slices and μCP as evidenced by the DAPI-stained cells of a slice superimposing a μCP. To test the neuronal growth of cholinergic neurons, a brain slice containing a region of nBM was placed onto a NGF μCP. Our analysis clearly showed selective ChAT+ outgrowths from the slice along the NGF printed regions. Their appearance, however, was very diverse, ranging from single nerve fibers and nerve fiber bundles to thick nerve fiber networks. The diversity of outgrowths may result from the composition of the slice itself, depending on which cells and factors are more highly enriched along the NGF μCP at the beginning of the culture. We found not only outgrowths but also a ChAT+ fiber reorganization along the NGF μCP within the brain slice, pointing toward a highly adaptable system. Moreover, we observed ChAT+ cells and, occasionally, an area of cells with unknown origins which migrated along the NGF μCP as well. This underscores the importance of NGF for cholinergic neurons, but also that NGF appears to affect other neuronal cell mechanisms as well. The neuronal marker MAP-2 co-localized with the ChAT+ processes, which strengthens the assumption of neuronal growth along NGF μCP. Additional immunohistochemical staining using p75^NTR^ antibodies showed strong colocalization with ChAT+ processes providing additional evidence that cholinergic neurons grew along the NGF μCP. No co-staining was seen for astroglial GFAP, microglial Iba-1, and vascular laminin. It is likely that the collagen *per se* provides a potent substrate to stimulate growth along the microprints. The ChAT+ nerve fibers grew ~280 μm out of the brain slices after 3 weeks but did not extend further after 6 weeks. It was assumed that the collagen degrades completely within 14 days, but the outgrowing processes survive on the membrane for longer periods. No more growth was seen after 3 weeks, probably due to the lack of NGF microprints. To our knowledge, our study is the first to observe neuronal growth along NGF μCP in slices.

### Intra-Slice Re-organization on NGF Microcontact Prints

For technical reasons, the slices were positioned at the center of the μCP as this was easier and guaranteed that the slices were clearly connected to the μCP. Thus, this strategy enabled us not only to visualize outgrowth but also to study any internal reorganization within the slice along the NGF μCP. The most interesting observation was the strong astrocytic (GFAP+) internal reorganization along the NGF μCP after 3 weeks in culture. Furthermore, our analysis showed that in some parts, cholinergic (ChAT+) internal reorganization took place along the NGF μCP. In contrast, no vessel (Laminin+) and microglial (Iba-1+) internal reorganization were visible. The reasons for the NGF-mediated re-organization of astrocytes are entirely ambiguous. There is evidence that NGF is produced and released by astrocytes after inflammation, suggesting an autocrine or paracrine mechanism (50–52). Further, NGF induces the expression of the p75^NTR^ receptor in astrocytes especially during development, inflammation, and after injury (50, 53). More importantly, it has been reported that NGF facilitates astrocytic migration *via* the p75^NTR^ as the trkA receptor is not expressed in astrocytes (53, 54). In fact, NGF can also increase the migration of multipotent astrocytic stem cells (55), but also possibly of oligodendroglia expressing p75^NTR^ and associated with radially oriented astroglia (56). The process of astrocytic migration is unclear, but it seems to be particularly essential for the structural organization during development or scar formation after brain damage (57). Our data provide evidence of the self-reorganization of astrocytes along the NGF μCP, considering that NGF may be a strong chemoattractive growth factor for astrocytes. However, we cannot exclude that collagen alone or in combination with NGF is responsible for the self-reorganization of the astrocytes. It has been seen that astrocytes interacted and aligned with the μCP of laminin (58). The self-reorganization of the astrocytes may also force cholinergic (ChAT+) cells to migrate and organize along the NGF μCP (or the opposite way round).

### Migration of Cells Along the μCP Lanes

In our study, we observed some migration of cells. It seems possible that cholinergic ChAT+ neurons migrated along the μCP(NGF) lanes. It seems very likely that NGF supports some form of cellular migration, although this mechanism is not fully understood. Especially, it is novel to see the migration of fully differentiated postnatal cholinergic neurons. We further showed that endothelial cells or microglia migrated along the collagen μCP. On the other hand, we can exclude migration of astroglia, as we did not see any staining along the μCP lanes. Regarding microglia and endothelial cells, we do not suggest an NGF-dependent process. Rather, it is possible that the carrier material collagen strongly supports the differentiation, attachment, migration, proliferation, and survival of those cells (39). In fact, it has been well-documented that extracellular matrix proteins, such as poly-lysine, fibronectin, and laminin, appear to initiate similar behavior in different cells types (44–46). Astrocytes do not migrate or extend processes along the μCP, which could be explained by the (a) downregulated GFAP expression in those areas, (b) different subtypes of astrocytes that do not express GFAP, or (c) a switch from reactive to inactive state and degradation of GFAP. Definitely, GFAP represents the reactive astrocytes within the slices, which are more pronounced at the borders. Alternatively, GFAP+ cells may need a longer time in culture to migrate the same distance as other cells (45). More work is necessary to study the migratory capacity along the μCP.

### Limits of the Study and Outlook

This work has some limits: (a) Collagen is a potent biomaterial, but we observed some minor changes in the composition of the hydrogels, which could influence the results. Collagen degrades over time and releases NGF within 2 weeks, thus it seems likely that the outgrowth along NGF μCP occurred within 2 weeks. (b) In our study, we have not performed retrograde tracing experiments. So, it would be interesting to apply a fluorescent dye and to observe if it is retrogradely transported to the slices, such a dye could be MiniRuby. (c) In this respect, the live-cell imaging of such tracing could also be of interest to observe the retrograde transport of the fluorescent dye. (d) Although we found a relatively good outgrowth of ~300 μm over 3–6 weeks, it is not clear if the outgrowth is time-dependent and can be extended. Partly it was hard to differentiate the border of the brain slices and to measure the distance length. (e) In our experiments we used NGF in the medium to prime the initial survival of cholinergic neurons; we have not tested if the survival on μCP(NGF) alone is sufficient. In addition, the dissection of the nBM brain area was not easy and it can be possible to collect and culture slices that do not contain cholinergic neurons. To enhance the chance, two brain slices were placed on a membrane. The quality of the slices was always visualized optically and a good flattening and high transparency are an indicator of a good slice preparation. Thicker slices and slices without any cholinergic neurons were deleted from this study.

The method of μCP in combination with organotypic brain slices is very potent and promising and has several future applications: (a) First, principally we can microprint any protein or peptide of interest (in preliminary experiments we microprinted tau and Aβ, Supplementary Figure 1). This increases the potential of the method markedly, as any commercial protein or peptide can be loaded. (b) Such a broad range may also allow us to microprint a cocktail of different proteins or peptides at the same time. (c) The method of μCP may also allow the printing of different patterns in different directions, which again markedly enhances the possibilities to study neuronal fiber growth. (d) Although not tested, we may also consider printing cells, such as isolated astrocytes or stem cells. (e) This method of μCP may also allow us to study vessels and their arborization out of the brain slices and the possible re-connection of vessels. (f). This method of μCP may have the potential to induce the migration of microglial cells along a printed line with small 1.0 μm beads which can be incorporated. (g) The use of different biomaterials may potentiate the μCP pattern and may further improve the method. (h) Such a method may be useful to study the neuronal re-growth of cholinergic neurons in AD, but also of dopaminergic neurons in Parkinson's disease. Alternatively, the re-growth and connection of spinal cord nerve fibers may also be studied. (i) Finally, this may markedly reduce animal experiments as many slices (depending on thickness and brain region of interest) can be prepared from one brain contributing to the 3Rs.

In summary, we showed for the first time that we can microprint NGF onto 0.4 μm semipermeable membranes using collagen as a loading vehicle. The cholinergic nBM organotypic brain slices survived and the cholinergic nerve fibers extended toward the NGF μCP. We also showed that GFAP+ astrocytes re-organized in the slices on the NGF μCP. We showed here for the first time that we can link μCP with brain slices and this method has a high capability to study different aspects in neurobiology. As this technique is easy, fast, and cheap, we can print nearly any protein/peptide onto a membrane. This technique will allow studying nerve fiber growth but may also be suitable to study vessel growth or migration of cells.

## Data Availability Statement

The raw data supporting the conclusions of this article will be made available by the authors, without undue reservation.

## Ethics Statement

Ethical review and approval was not required for the animal study because all experiments conformed to Austrian guidelines on the ethical use of animals and were in line with the 3Rs rule as all efforts were made to reduce the number of animals. All animal experiments were defined as Organentnahme according to Austrian laws.

## Author Contributions

KS performed all experiments and wrote the manuscript. CH designed and analyzed the data and wrote the manuscript. Both authors contributed to the article and approved the submitted version.

## Funding

This study was supported by the Austrian Science Funds (P32558-B).

## Conflict of Interest

The authors declare that the research was conducted in the absence of any commercial or financial relationships that could be construed as a potential conflict of interest.

## Publisher's Note

All claims expressed in this article are solely those of the authors and do not necessarily represent those of their affiliated organizations, or those of the publisher, the editors and the reviewers. Any product that may be evaluated in this article, or claim that may be made by its manufacturer, is not guaranteed or endorsed by the publisher.
