# Supplementary figures and images for: Microcontact Printing of Cholinergic Neurons in Organotypic Brain Slices
Source: Front Neurol. 2021 Nov 17;12:775621. doi: 10.3389/fneur.2021.775621 (PMC8636044; doi:10.3389/fneur.2021.775621)

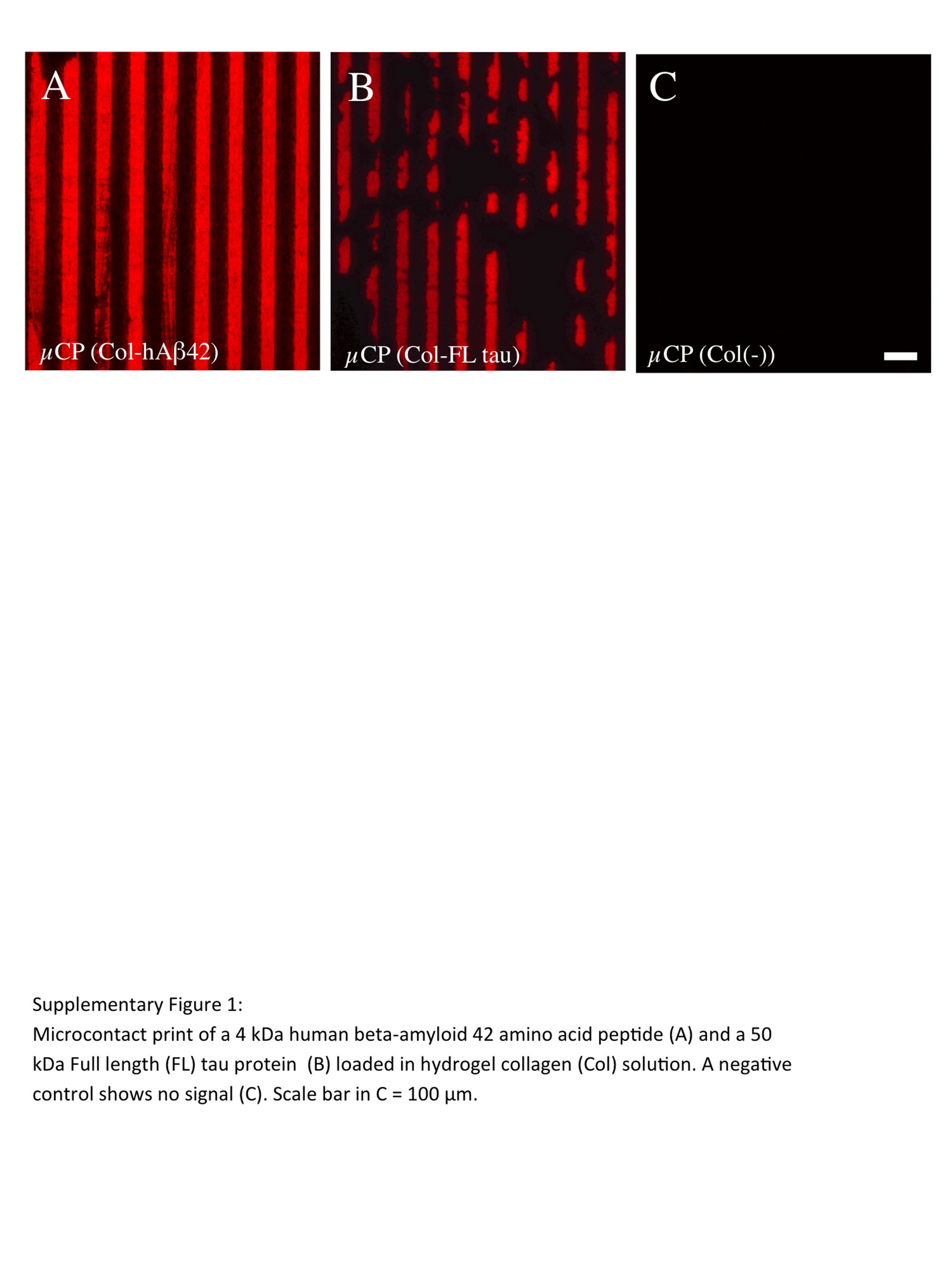

Supplement: Supplementary file 1 [file Image_1.JPEG]
